# Sensor-Informed Finite Element Model Updating of a Railway Bridge Using Hybrid Differential Evolution–Levenberg–Marquardt Optimisation: A Case Study

**DOI:** 10.3390/s26216948

**Published:** 2026-11-03

**Authors:** Hoang N. Bui, Jose C. Matos, Son N. Dang, Nhung T. C. Nguyen

**Affiliations:** 1 School of Engineering and Applied Sciences, State University of New York at Buffalo, New York, NY 14360, USA; 2 Department of Civil Engineering, ISISE, ARISE, University of Minho, 4800-058 Guimarães, Portugal; 3 Faculty of Civil Engineering, University of Transport and Communications, Hanoi 100000, Vietnam

**Keywords:** digital twin, finite element model updating, differential evolution, Levenberg–Marquardt, LVDT, urban railway bridge, displacement monitoring

## Abstract

Field displacement measurements can support FE model updating, although the resulting model remains conditional on the available observations and modeling assumptions. This study proposes a hybrid Differential Evolution–Levenberg–Marquardt (DE–LM) framework for updating an FE model using train-induced displacement histories measured by linear variable differential transformers (LVDTs). A prestressed-concrete box-girder railway bridge was investigated, with the numerical model explicitly representing the box girder, sleepers, rails, and equivalent rail-fastening connections. Preliminary parameter screening was conducted to select three influential parameters: the box-girder elastic modulus and the effective vertical and lateral fastening stiffnesses. DE was employed to explore the bounded parameter space, after which LM refined the best global solution using local response sensitivities. Physical parameter bounds and regularisation were incorporated to limit non-unique and unrealistic solutions. Complete train passages were separated into calibration and independent validation datasets. The hybrid procedure reduced the initial objective value by approximately 90.5% and produced physically admissible parameter changes. For the independent validation dataset, the updated model achieved a mean normalized root mean square error of 2.17%, compared with 3.07% for DE-only, 4.94% for LM-only, and 7.46% for the initial model. The mean peak-displacement error was reduced to 0.70%. For the investigated case, the selected hybrid solution achieved lower validation errors than the reported DE-only and nominal-start LM solutions. The calibrated model provides a physics-based foundation for subsequent railway-bridge digital-twin development.

## 1. Introduction

Urban railway bridges are subjected to repeated train passages, environmental variations, progressive material deterioration, and possible changes in support and connection conditions [1,2]. Reliable numerical models are therefore required to evaluate their structural response and support condition assessment throughout their service life. In this context, the digital twin concept provides a framework for connecting a physical structure with its virtual representation through measurement data [3]. Nevertheless, the effectiveness of such a framework depends strongly on the accuracy of its baseline finite element (FE) model [4,5]. A model constructed exclusively from design drawings and nominal material properties may not adequately represent the as-built structure because of uncertainties in material characteristics, structural dimensions, boundary conditions, connection behaviour, and operational loading [6]. Establishing a physically representative baseline virtual model through measurement-informed FE model updating is consequently a necessary step toward developing a reliable bridge digital twin [7].

Finite element model updating aims to reduce discrepancies between measured structural responses and the corresponding numerical predictions by adjusting selected uncertain model parameters [8,9]. Existing approaches can generally be divided into response-surface, Bayesian, sensitivity-based, and population-based optimization methods [10,11]. Sensitivity-based techniques update model parameters iteratively using derivatives of the selected residuals with respect to those parameters [12]. These techniques are computationally efficient when the initial model is close to the actual structural state, and the objective function is sufficiently smooth. They have therefore been widely employed for updating material properties, stiffness distributions, masses, and boundary conditions [13,14]. However, their performance can deteriorate when the inverse problem is highly nonlinear, poorly conditioned, or affected by correlated parameters. Their convergence may also depend strongly on the initial parameter values, potentially leading to physically implausible solutions or convergence to a local minimum.

Population-based metaheuristic algorithms provide an alternative because they explore a broader parameter space without requiring analytical gradients [15]. Genetic algorithms, particle swarm optimisation, differential evolution, simulated annealing, and their variants have been applied to structural model updating and damage identification [16]. These methods are attractive for problems involving nonlinear responses, multiple local minima, discontinuous variables, and complex parameter constraints. However, global optimization algorithms typically require numerous FE evaluations and may converge slowly when approaching the optimum. They can identify a promising region of the parameter space but are often less efficient in obtaining a highly accurate final solution. Conversely, local least-squares algorithms can converge rapidly near a solution but remain sensitive to the starting point [17,18]. This complementary behaviour provides a clear motivation for combining global exploration with local refinement in a hybrid model-updating strategy.

Differential Evolution (DE) [19,20] is a population-based evolutionary algorithm that generates candidate solutions using scaled differences between population members. Its mutation and crossover mechanisms enable effective exploration while requiring relatively few algorithm-specific control parameters. DE has been used in structural optimization and FE model updating because it can accommodate bounded parameters, nonlinear objective functions, and multimodal search spaces [21]. Nevertheless, conventional DE may lose population diversity during the later stages of optimisation, resulting in slow convergence or stagnation around a near-optimal solution. Increasing the number of generations can improve the final solution but also substantially increases the computational cost when each objective-function evaluation requires an FE analysis.

The Levenberg–Marquardt (LM) algorithm combines the characteristics of the gradient-descent and Gauss–Newton methods for solving nonlinear least-squares problems [22,23]. When initialised within a suitable neighbourhood of the optimum, LM can reduce response residuals efficiently and provide accurate parameter estimates. This property makes it suitable for the local refinement stage of FE model updating. However, LM alone is not appropriate for broad exploration because the initial parameter vector, parameter scaling, measurement noise, and the conditioning of the sensitivity matrix can strongly influence its solution. A hybrid DE–LM strategy can exploit the global search capability of DE to locate promising parameter regions and subsequently employ LM to refine the best candidates. The two algorithms therefore perform distinct rather than duplicated functions: DE reduces dependence on the initial model, while LM improves local convergence and solution precision.

Most bridge model-updating studies have relied on modal characteristics, including natural frequencies, mode shapes, and modal assurance criteria, because such features provide information about the global dynamic behaviour of a structure [24]. Acceleration-based modal data are particularly common in structural health monitoring [25]. However, modal identification introduces additional uncertainties related to measurement noise, excitation conditions, sensor placement, modal-order selection, and the adopted system-identification method. Moreover, natural frequencies alone may be insufficient to identify multiple uncertain parameters because different parameter combinations can produce similar frequency changes. These difficulties become particularly relevant for operational railway bridges, where train-induced responses are non-stationary and may vary with axle load, train speed, track irregularity, and environmental conditions.

Displacement measurements offer a direct representation of structural deformation and can be highly informative for parameters governing flexural stiffness, bearing restraint, connection behaviour, and load transfer [26]. Linear variable differential transformers (LVDTs) provide high-resolution displacement measurements and are commonly used in laboratory tests, controlled load tests, and short-term bridge monitoring campaigns [27]. Compared with acceleration measurements, LVDT data do not require numerical integration to obtain displacement and are therefore less affected by integration drift. Nevertheless, their use in full-scale bridge model updating remains challenging [28]. LVDTs measure responses only at a limited number of accessible locations and normally require a stable reference point. The resulting inverse problem may therefore be underdetermined, particularly when the number of uncertain model parameters is large. In addition, fitting only the maximum displacement can conceal discrepancies in other parts of the measured response. Train-induced displacement histories have been used for time-domain FE model updating of railway bridges [29]. The present study adopts this response-based approach using relative rail–girder displacement measurements.

Parameter identifiability is another important issue. Increasing the number of updated parameters does not necessarily improve model fidelity because weakly sensitive or highly correlated parameters cannot be uniquely estimated from limited measurements. Without appropriate controls, optimization may achieve a low response error through compensating parameter changes that have little physical meaning. Sensitivity analysis, parameter bounds, regularisation, and independent validation should therefore be incorporated into the updating process. In the present study, separate train passages are used for calibration and validation. The withheld passages assess prediction beyond the records used for parameter estimation.

Combining global exploration with local refinement is an established strategy in FE model updating. Teughels et al. [30] developed coupled local minimizers for updating structural models using experimental modal data. Jung and Kim [31] combined a genetic algorithm with simplex-based local optimization to update a prestressed-concrete bridge using natural frequencies, mode shapes, and static displacements. Displacement-history-based updating of railway bridges has also been investigated, including the train-induced response approach presented by Feng and Feng (2015) [29]. These studies establish that neither global–local hybridisation nor the use of displacement histories alone constitutes a new model-updating concept.

Building on these approaches, the present study develops a displacement-based DE–LM implementation for an operational urban railway bridge in which the monitored rail response depends on both girder stiffness and local fastening behaviour. The formulation combines separately normalized vertical and lateral LVDT displacement histories to update the box-girder elastic modulus and effective fastening stiffnesses. Physical bounds and regularisation restrict admissible parameter changes, while a progress-based switching rule transfers the best DE solution to LM for local refinement. Complete train-passage events are assigned to separate calibration and validation groups to assess prediction beyond the records used for parameter estimation.

The contribution therefore lies in the integration and field evaluation of these elements for the investigated rail–bridge system. Specifically, the study: (i) formulates a joint displacement-history objective for updating girder and fastening parameters; (ii) implements a DE-to-LM transition governed by the observed progress of the global search; and (iii) evaluates the updated model using withheld train passages and comparisons with DE-only and LM-only procedures. These comparisons assess the benefit of hybridisation within the present application; they do not establish general superiority over other global–local methods. The resulting model is intended as a calibrated numerical foundation for subsequent digital-twin development.

The remainder of the paper is organised as follows. Section 2 presents the LVDT-based model-updating formulation, objective function, and hybrid DE–LM procedure. Section 3 describes the investigated bridge structure, LVDT measurement programme, initial FE model, and parameter-selection process. Section 4 presents and discusses the updating, validation, and comparative results. Finally, Section 5 summarises the main findings, limitations, and future research directions.

## 2. LVDT-Based Hybrid DE–LM Model-Updating Framework

### 2.1. Model-Updating Formulation and LVDT-Based Objective Function

The initial finite element model is constructed using the available design information, nominal material properties, structural dimensions, and assumed boundary conditions [32]. However, differences between the nominal and actual properties of the bridge may lead to discrepancies between the measured and numerical responses. Finite element model updating is therefore formulated as an inverse problem in which selected uncertain parameters are adjusted to minimise the difference between the LVDT measurements and the corresponding FE predictions. The vector of updating parameters is expressed as:

(1) $$\theta =\;{\left[\theta_{1},\theta_{2},\ldots ,\theta_{n_{p}}\right]}^{T},\;\;\theta^{L}\;\leq \;\theta \;\leq \;\theta^{U}.,$$

where *n_p_* is the number of updating parameters, while **θ***^L^* and **θ***^U^* denote their lower and upper bounds, respectively. Depending on the initial modelling uncertainties, the parameters may include the elastic modulus, material density, non-structural mass, bearing stiffness, connection stiffness, or other quantities governing the displacement response. The parameter bounds are defined using the available design information, engineering judgement, and physically admissible ranges. These constraints prevent the optimization process from achieving a low response error through unrealistic parameter combinations.

Let $u_{i,l}^{m}(t_{j})$ denote the displacement measured by LVDT *i* at time *t_j_* during loading event *l*, and let $u_{i,l}^{\mathrm{FE}}(t_{j},\theta )$ be the corresponding displacement predicted by the FE model. The measured displacement represents rail motion relative to the girder-mounted LVDT reference. The corresponding FE response is therefore defined as the displacement difference between the rail contact point and the mounting-reference point, projected onto the measurement direction. The normalized displacement residual is defined as

(2) $$r_{i,l,j}(\theta )=\frac{u_{i,l}^{\mathrm{FE}}(t_{j},\theta )\;-\;u_{i,l}^{m}(t_{j})}{s_{i,l}},$$

where *s_i_*_,_*_l_* is a scale factor introduced to prevent measurements with larger displacement amplitudes from dominating the updating process. In the present formulation, *s_i_*_,_*_l_* can be selected as the maximum absolute displacement measured by sensor *i* during loading event *l.* A small positive constant should be used when the measured response amplitude is close to zero. The displacement-history component of the objective function is formulated as the weighted mean square of the normalized residuals:

(3) $$J_{\mathrm{hist}}(\theta )\;=\;\frac{1}{N_{r}}\sum_{l=1}^{N_{L}}\sum_{i=1}^{N_{S}}\sum_{j=1}^{N_{T}}w_{i,l}{\left[r_{i,l,j}(\theta )\right]}^{2},$$

where *N_L_*, *N_S_*, and *N_T_* are the numbers of loading events, LVDT measurement locations, and selected time samples, respectively; *N_r_* = *N_L_N_S_N_T_* is the total number of displacement residuals; and *w_i_*_,_*_l_* is the weight assigned to the corresponding measurement series. Equal weights may be used when the LVDTs have comparable measurement quality. Alternatively, lower weights can be assigned to records affected by higher noise, incomplete data, unstable reference conditions, or other identified measurement uncertainties.

Using the complete displacement histories retains more information than updating the model solely against maximum displacement values. In particular, it allows the objective function to consider the evolution of the structural response as the train passes through the investigated span. Nevertheless, the recorded data may contain a large number of highly correlated samples. The signals can therefore be downsampled or represented by selected response points after filtering, baseline correction, and synchronisation with the loading events. The same preprocessing procedure must be applied to both the measured and simulated responses to ensure a consistent comparison.

Because different combinations of structural parameters may produce similar displacement responses, the updating problem can be non-unique. A regularisation term is introduced to discourage excessive deviations from the initial parameter estimates:

(4) $$J_{\mathrm{reg}}(\theta )\;=\;\frac{1}{n_{p}}\sum_{k=1}^{n_{p}}{\left(\frac{\theta_{k}-\theta_{k}^{0}}{\theta_{k}^{U}-\theta_{k}^{L}}\right)}^{2},$$

where $\theta_{k}^{0}$ is the initial value of parameter k. This term does not force the updated parameters to remain close to their nominal values; rather, it penalises unnecessarily large changes when several parameter combinations provide similar agreement with the measurements. The overall model-updating problem is consequently expressed as

(5) $$\theta^{*}=\underset{\theta^{L}\leq \theta \leq \theta^{U}}{\mathrm{argmin}}\left[J(\theta )\right],\;\;J(\theta )\;=\;J_{\mathrm{hist}}(\theta )\;+\;\lambda J_{\mathrm{reg}}(\theta ).,$$

where θ^*^ is the updated parameter vector and *λ* controls the influence of regularisation. A small value of *λ* allows the measured response to dominate the updating process, whereas an excessively large value may prevent the model from departing sufficiently from its initial state. Its value should therefore be selected through preliminary analysis or validation using measurements not included in the calibration dataset.

The objective function in Equation (5) is compatible with both stages of the proposed hybrid optimization framework. DE explores the bounded parameter space using the scalar objective value *J*(**θ**), whereas LM locally minimises the associated residual vector after DE has identified a promising parameter region. The calibration dataset is used to estimate θ^*^, while separate LVDT records are retained for independent validation. This separation is necessary to determine whether the updated FE model improves the prediction of structural responses beyond the measurements used directly in the optimisation.

### 2.2. Differential Evolution and Levenberg–Marquardt Algorithms

The proposed model-updating framework combines Differential Evolution (DE) and the Levenberg–Marquardt (LM) algorithm because they provide complementary search capabilities. DE performs a population-based global search without requiring gradient information, whereas LM uses local response sensitivities to refine a promising solution. This section presents the main operations of the two algorithms, while their hybridisation is described in Section 2.3.

DE represents each candidate solution as a vector containing the FE updating parameters. At generation g, the i-th individual is written as

(6) $$\theta_{i}^{g}\;=\;{\left[\theta_{i,1}^{g},\theta_{i,2}^{g},\ldots ,\theta_{i,n_{p}}^{g}\right]}^{T},\;\;i\;=\;1,2,\ldots ,N_{P},$$

where *N_P_* is the population size and *n_p_* is the number of updating parameters. The initial population is randomly generated within the lower and upper bounds defined in Equation (1).

In the mutation stage, the commonly used strategy generates a mutant vector as:

(7) $$v_{i}^{g}\;=\;\theta_{r_{1}}^{g}\;+\;F\left(\theta_{r_{2}}^{g}\;-\;\theta_{r_{3}}^{g}\right),$$

where *r_1_, r_2_*, and *r_3_* are mutually different indices selected randomly from the population and are also different from *i*. The mutation factor *F* controls the magnitude of the differential variation and therefore affects the balance between population diversity and search stability. Any component of the mutant vector that violates its prescribed physical bounds is returned to the admissible parameter domain before the corresponding FE analysis is conducted.

The mutant vector is subsequently combined with the current target vector through binomial crossover:

(8) $$z_{i,k}^{g}\;=\;\left\{\begin{array}{ll} v_{i,k}^{g}, & \mathrm{if}\;{\mathrm{rand}}_{k}\;\leq \;C_{R}\;\mathrm{or}\;k\;=\;k_{\mathrm{rand}}\\ \theta_{i,k}^{g}, & \mathrm{otherwise}. \end{array}\right.,$$

where $z_{i,k}^{g}$ and $v_{i,k}^{g}$ are the k-th components of the trial and mutant vectors, respectively; C_R_ is the crossover probability; and *k*_rand_ ensures that at least one component of the trial vector is inherited from the mutant vector.

The trial vector competes with the target vector according to the objective function defined in Equation (5):

(9) $$\theta_{i}^{g+1}=\left\{\begin{array}{ll} z_{i}^{g}, & J\left(z_{i}^{g}\right)\leq J\left(\theta_{i}^{g}\right),\\ \theta_{i}^{g}, & \mathrm{otherwise}. \end{array}\right.,$$

This greedy selection preserves solutions that improve or maintain the objective value. Through repeated mutation, crossover, and selection, the DE population explores different regions of the bounded parameter space. The differential mutation mechanism is particularly suitable for the present problem because the parameter scales and interactions are not necessarily known in advance. Nevertheless, as the population becomes increasingly concentrated, the differential variations become smaller, and the improvement in the objective function may slow considerably.

The LM algorithm is designed for nonlinear least-squares problems and combines the rapid local convergence of the Gauss–Newton method with the stability of gradient descent. In the present study, the residuals defined in Equation (2) are assembled into the residual vector **r**(**θ***^q^*). The LM parameter update at local iteration q is expressed as

(10) $$\theta^{q+1}\;=\;\theta^{q}\;-\;{\left[S_{q}^{T}S_{q}\;+\;\mu_{q}I\right]}^{-1}S_{q}^{T}r\left(\theta^{q}\right),$$

where **S***_q_* is the sensitivity matrix of the residual vector with respect to the updating parameters, **I** is the identity matrix, and *μ_q_* is the damping parameter. Each element of the sensitivity matrix can be approximated using finite differences:

(11) $$S_{a,k}\;\approx \;\frac{r_{a}\left(\theta^{q}\;+\;\Delta \theta_{k}e_{k}\right)\;-\;r_{a}\left(\theta^{q}\right)}{\Delta \theta_{k}}.,$$

where *r_a_* is the a-th residual, Δ*θ_k_* is a small perturbation applied to parameter k, and **e***_k_* is the corresponding unit vector. The perturbation should be sufficiently small to represent local sensitivity while remaining large enough to avoid numerical errors in the FE response.

The damping parameter controls the behaviour of LM. A relatively large value of *μ_q_* produces a conservative gradient-descent-like step, whereas a small value results in a Gauss–Newton-like step with faster local convergence. If the proposed update reduces the objective function, the step is accepted, and the damping parameter is decreased. Otherwise, the update is rejected and *μ_q_* is increased. Updated parameters are also constrained within their prescribed physical bounds.

LM can accurately refine a parameter vector when initialised within the attraction region of a suitable minimum. However, when applied directly to the initial FE model, it may converge to a local solution or become unstable because of parameter correlation and limited LVDT information. DE is therefore used to provide a robust starting point for LM. The criterion for transferring the solution between the two algorithms and the complete computational workflow are presented in the following section.

### 2.3. Hybrid DE–LM Strategy and Computational Procedure

**Figure 1:**
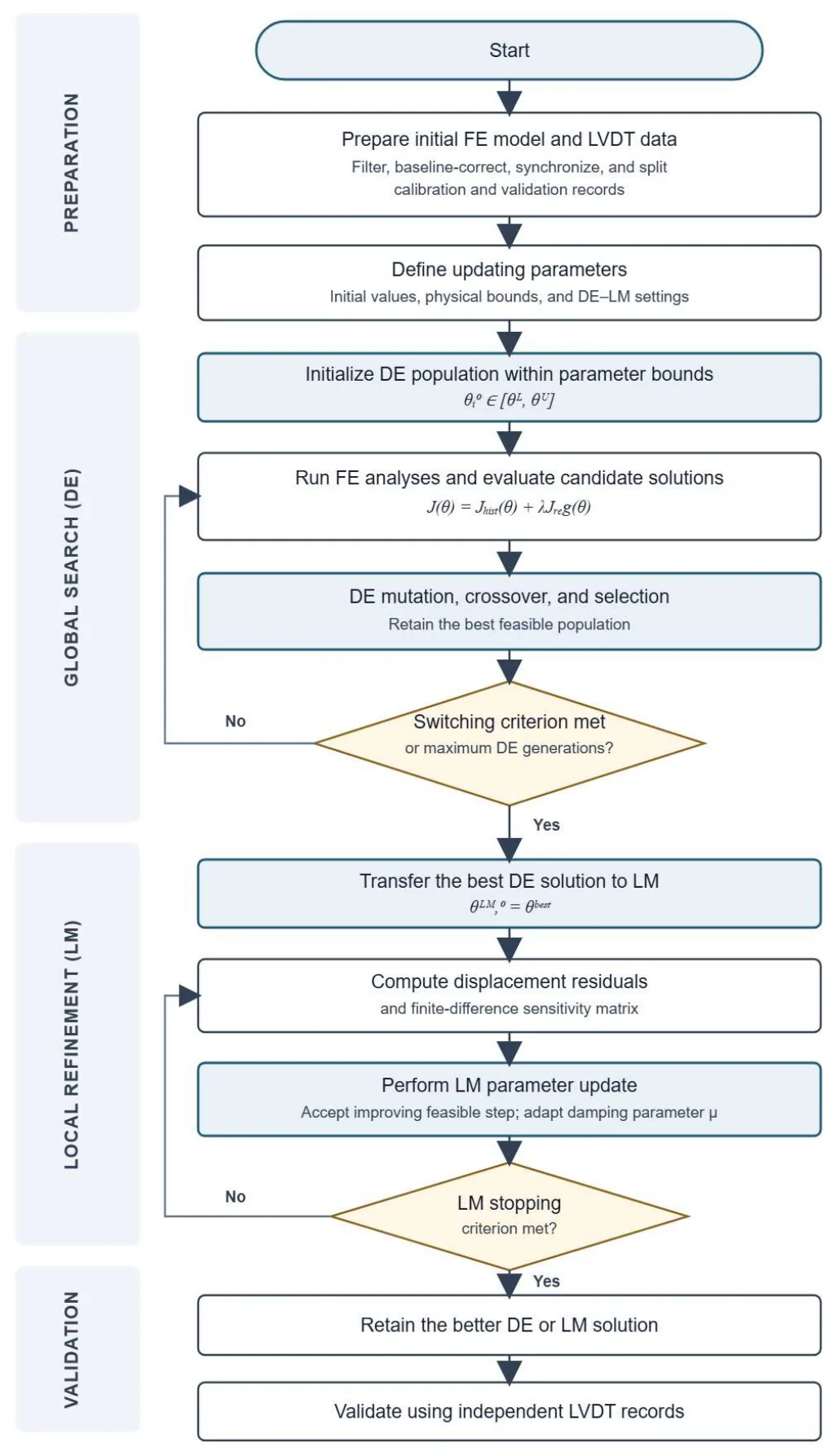
Computational workflow of the proposed hybrid DE–LM framework for LVDT-based finite element model updating.

The proposed hybrid strategy sequentially combines the global exploration capability of DE with the local refinement efficiency of the LM algorithm. As illustrated in Figure 1, the computational procedure consists of four main phases: model and data preparation, global optimization using DE, local refinement using LM, and independent validation. The two algorithms are assigned distinct roles to avoid redundant computation: DE identifies a promising region within the bounded parameter space, whereas LM improves the accuracy of the best solution obtained from the global search.

The procedure begins with the preparation of the initial FE model and the measured LVDT responses. The displacement records are filtered, baseline-corrected, and synchronised with the corresponding loading events. The available measurements are then divided into calibration and validation datasets. Only the calibration records are used in the optimisation, while the remaining records are retained for an independent assessment of the updated model. The uncertain model parameters are subsequently defined together with their initial values and physically admissible lower and upper bounds. The DE and LM control parameters, convergence tolerances, and maximum numbers of iterations are also specified at this stage.

In the global-search phase, an initial DE population is randomly generated within the prescribed parameter bounds. Each population member represents a candidate set of FE model parameters. For every candidate, the FE model is evaluated, the numerical displacements are extracted at the LVDT measurement locations, and the objective function is calculated from the differences between the measured and simulated responses. Candidate solutions producing numerical instability or violating the physical constraints are rejected or assigned a sufficiently large penalty value.

The DE population is evolved through mutation, crossover, and selection. At each generation, trial solutions are compared with their corresponding target solutions, and those providing lower objective values are retained. This process maintains population diversity during the early stages while progressively directing the search toward parameter regions that provide better agreement with the LVDT measurements. The best feasible solution and its objective value are recorded after each generation.

The transition from DE to LM is controlled by the progress of the global search. DE continues while the best objective value exhibits meaningful improvement. When the improvement over a predefined number of generations falls below the specified tolerance, the global search is considered to have stagnated, and the best DE solution is transferred to LM. The transition is also performed when the maximum number of DE generations is reached. A minimum number of generations should be completed before LM is activated to prevent a premature switch before the parameter space has been adequately explored.

During the local-refinement phase, the best DE parameter vector is used as the initial LM solution. The displacement residuals and their sensitivities to the updating parameters are then evaluated. Because analytical sensitivities are generally unavailable for the adopted FE model, the sensitivity matrix is estimated by applying small finite-difference perturbations to individual parameters and repeating the numerical analysis.

Based on the calculated residuals and sensitivities, LM proposes a local parameter update. The new parameter vector is accepted only when it remains within the prescribed bounds and reduces the overall objective value. Following a successful update, the damping parameter is decreased to permit a faster Gauss–Newton-type search. If the proposed update does not improve the objective function, it is rejected, the previous solution is retained, and the damping parameter is increased to produce a more conservative subsequent step. The residuals and sensitivity matrix are then recalculated, and the local refinement continues.

The LM stage is terminated when the objective-function improvement becomes smaller than the prescribed tolerance, the parameter increment becomes sufficiently small, or the maximum number of local iterations is reached. The final LM solution is subsequently compared with the best solution obtained by DE. The solution associated with the lower objective value is retained as the final updated parameter vector. This comparison safeguards against possible deterioration during the local-search stage and ensures that hybridisation cannot produce a solution inferior to the best DE result.

Finally, the updated FE model is evaluated using the independent LVDT records excluded from calibration. Agreement between the predicted and measured displacement responses is assessed in terms of response histories, peak values, and overall prediction errors. Successful validation indicates that the updated parameters improve the predictive capability of the model rather than merely fitting the calibration data. The resulting model provides a measurement-informed baseline virtual representation of the investigated bridge, which can subsequently support data synchronisation and condition assessment within a digital-twin framework.

In this implementation, the transition between the two optimization stages is triggered by a reduction in the calibration objective over a prescribed monitoring window, subject to minimum and maximum DE generation limits. LM therefore starts from the best feasible DE solution when further global-search improvement becomes limited or the generation limit is reached. Both stages address the same bounded, regularized updating problem. The final solution is retained only if its objective value is no greater than that obtained before local refinement. These provisions specify the coordination of the two established algorithms for the present application.

For reproducibility, the population size, mutation factor, crossover probability, parameter bounds, switching criterion, convergence tolerances, finite-difference perturbations, damping-control strategy, random seeds, and maximum numbers of DE generations and LM iterations should be reported in the case-study section.

## 3. Case Study

### 3.1. Bridge Description and LVDT Monitoring Programme

**Figure 2:**
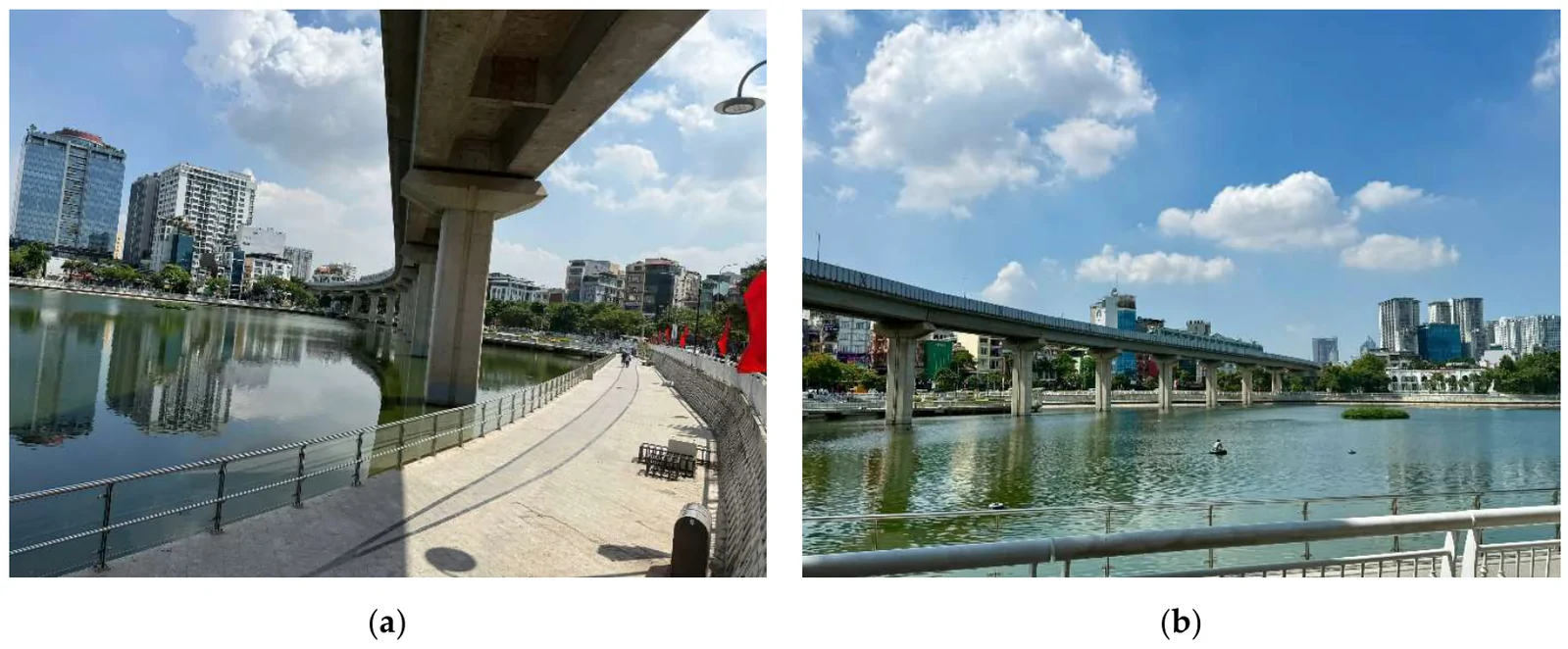
Investigated structure on the Cat Linh–Ha Dong urban railway line: (**a**) bottom view; (**b**) side view.

The investigated structure is located on the Cat Linh–Ha Dong urban railway line in Hanoi, Vietnam. The line is constructed entirely as an elevated railway and primarily consists of simply supported prestressed-concrete box-girder spans (Figure 2). The monitored span is located near Yen Nghia Station and represents a typical box-girder configuration adopted along the railway line. The structural system comprises the concrete box girder, reinforced-concrete sleepers, rails, rail pads, and fastening components. The railway track is a ballastless system in which the bridge deck directly supports the sleepers, while the rails are connected to the sleepers through elastic fastening systems. These components collectively govern the transfer of train-induced loads from the rails to the main bridge structure.

The operational train consists of four cars, with each car having a reported length of 19.52 m. The four-car train comprises 16 axles, which define the moving-load sequence adopted in the numerical model. The field measurements considered regular in-service train operations, with a representative operating speed of approximately 55.6 km/h. Recording the response under operational loading avoided the need for traffic interruption or a dedicated static load test and allowed the monitored structure to be evaluated under its actual service conditions.

A field monitoring programme was conducted using 4 S-Series Linear Variable Differential Transformer sensors with a measurement range of 0–50 mm. The measurement system also included a Solartron Metrology signal-conditioning and data-acquisition unit, signal cables, sensor-supporting frames, and a dedicated computer for data recording. The LVDTs were selected because they provide direct, high-resolution measurements of the rail displacement response generated by passing trains. Monitoring was conducted from 1 to 5 December 2022. Of the 55 recorded train passages, 12 were excluded because of noise, leaving 30 passages for calibration and 13 for validation. Recorded speeds ranged from 40 to 80 km/h. Temperature was 20–25 °C.

**Figure 3:**
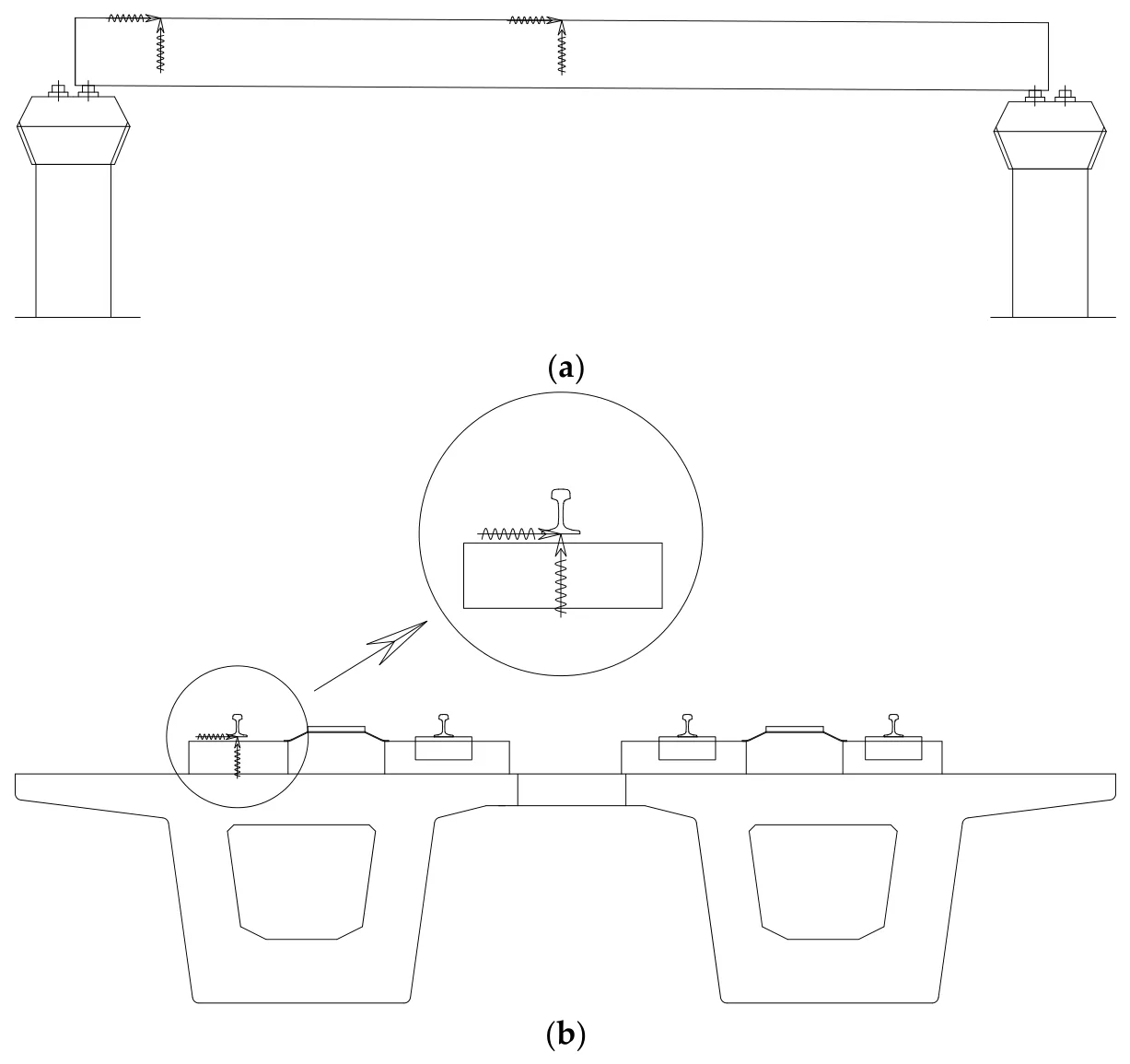
Field monitoring arrangement: (**a**) 1-span structural element; (**b**) cross-section.

**Figure 4:**
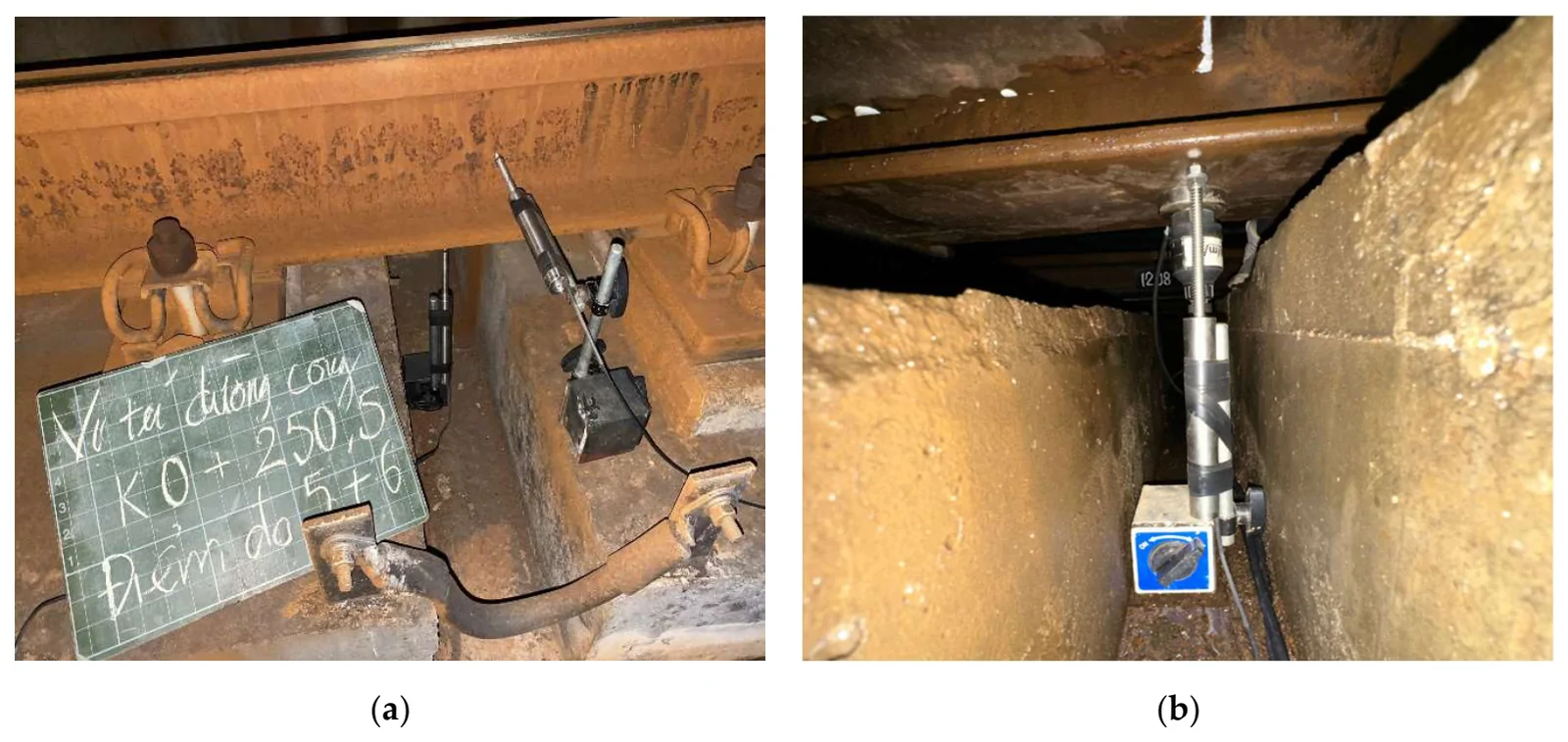
Girder-mounted LVDT arrangement for measuring relative rail–girder displacement: (**a**) lateral direction; (**b**) vertical direction.

Two representative measurement sections were considered (Figure 3 and Figure 4). The first was located approximately 5 m from the bridge support to capture the response near the end region of the span. The second was located at midspan, where the flexural response was expected to be more pronounced. The LVDT mounting frames were attached directly to the bridge girder, with the probes contacting the rails in the vertical and lateral directions. The recorded signals therefore represent rail displacement relative to the girder-mounted reference at each measurement location. They do not directly measure global girder deflection, because motion shared by the rail and the mounting reference is partly removed from the recorded response.

**Figure 5:**
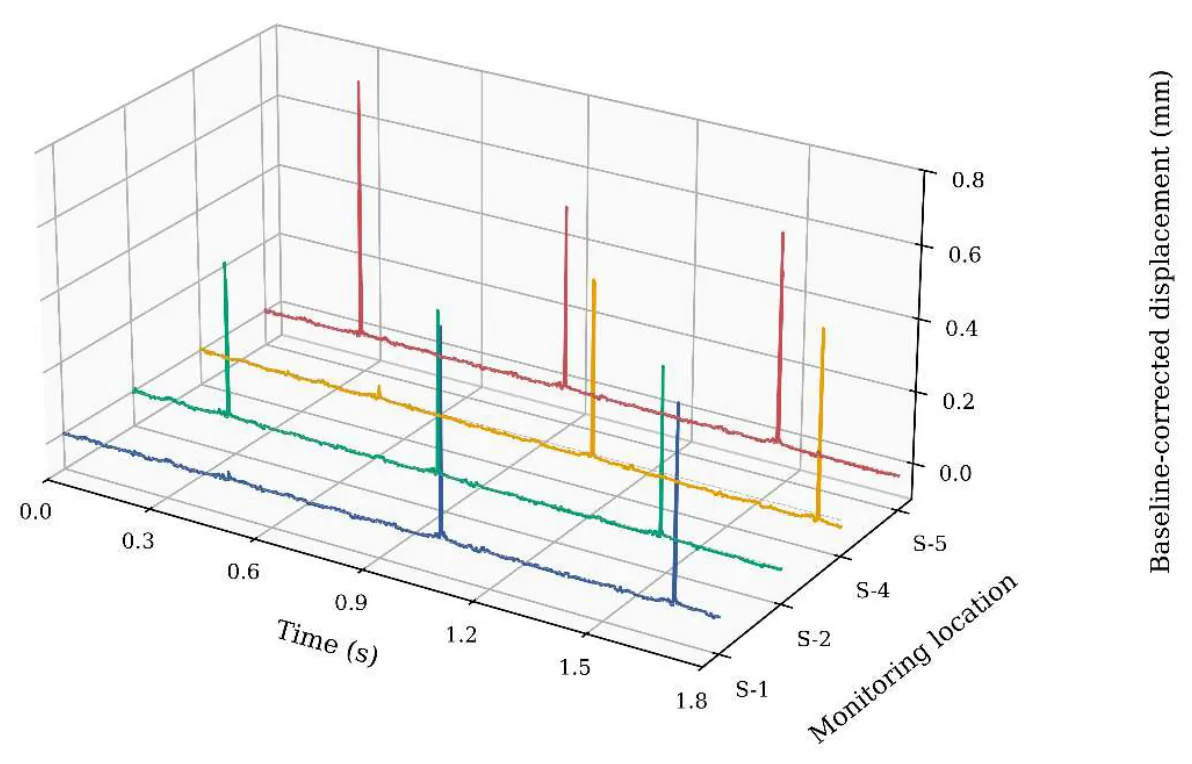
Selected segments of the measured relative rail–girder displacement records.

For each measurement event, the displacement signals were recorded continuously before the train entered the monitored span, throughout the complete train passage, and for a short period after the train left the span. The pre-passage and post-passage portions provided reference intervals for identifying the initial signal level and correcting possible baseline offsets. Figure 5 presents selected segments of the recorded responses. These segments do not cover the complete train passage and therefore do not display the full axle sequence.

The measured records were visually inspected before further analysis. Records affected by incomplete acquisition, evident sensor instability, or abnormal baseline variation were excluded. The retained signals were baseline-corrected and synchronised so that the measured and numerical histories represented the same train-passage interval (Figure 5). Because the sensors measured different response components and could exhibit different amplitude ranges, each record was subsequently treated using the normalisation procedure defined in Section 2.1. The LVDT signals were sampled at 200 Hz and filtered using a fourth-order, zero-phase Butterworth low-pass filter with a 20 Hz cutoff. The narrow peaks contained approximately 6–10 samples within their full width at half maximum.

The available records were divided into calibration and validation groups according to complete train passages rather than individual time samples. The calibration records were used to determine the FE model parameters through the proposed DE–LM procedure, whereas the validation records remained entirely excluded from the optimisation. This event-based separation reduces information leakage between the two datasets and provides a more meaningful assessment of whether the updated model can reproduce structural responses under independent train passages.

### 3.2. Initial Finite Element Model

**Figure 6:**
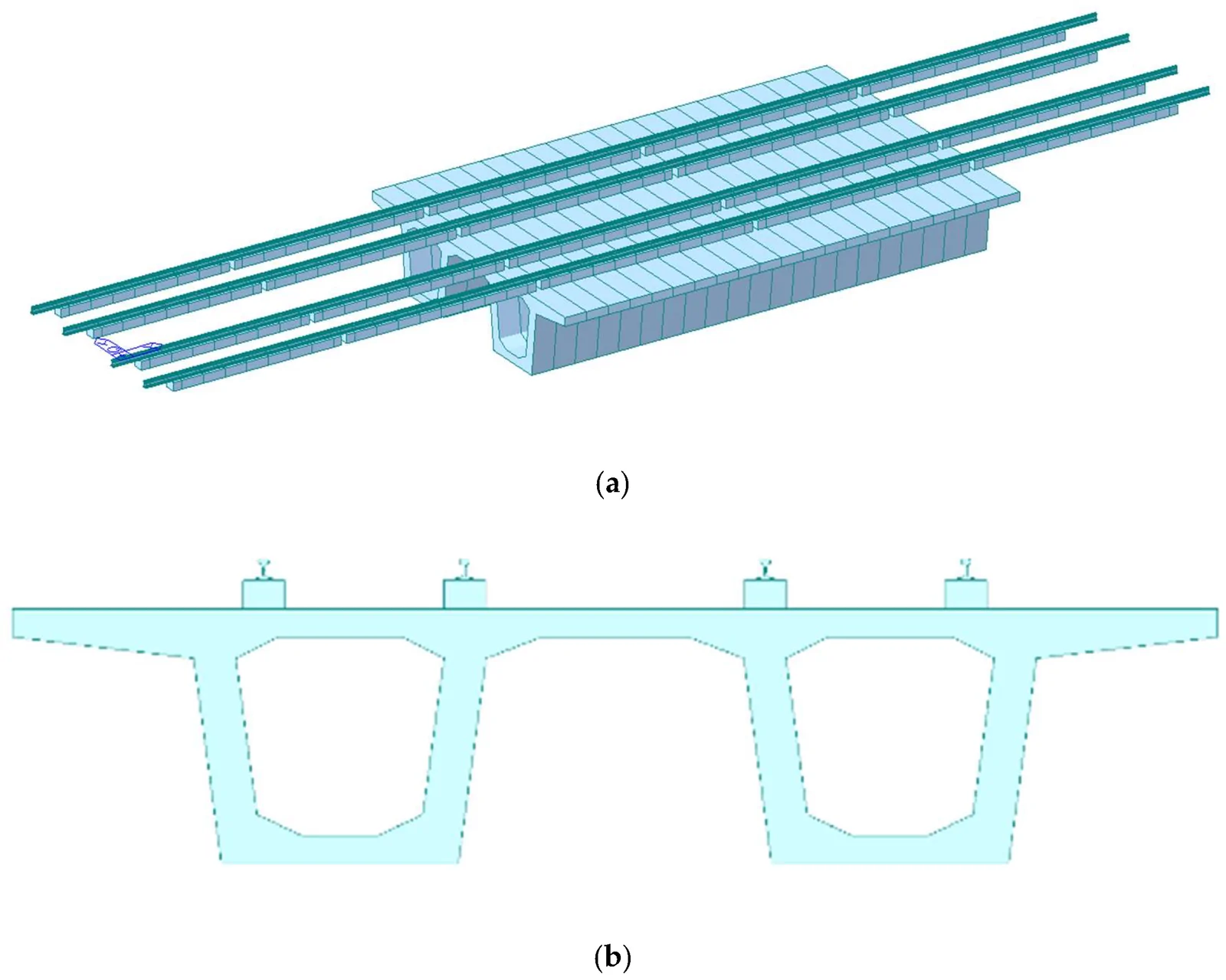
Initial finite element model of the investigated span: (**a**) entire model; (**b**) cross-section.

The initial finite element model was developed in MIDAS Civil using the available as-built information and nominal material properties of the investigated span (Figure 6). The purpose of this model was to reproduce the displacement response measured by the LVDTs under operational train loading and to provide the numerical basis for the subsequent DE–LM updating process. Since the sensors were installed on the rails, the model represented not only the main box girder but also the principal components governing the interaction between the track and bridge.

The numerical model comprised three main subsystems: the prestressed-concrete box girder, the reinforced-concrete sleepers, and the rails (Table 1) The investigated bridge span was represented as a simply supported concrete box girder with an effective modelled length of 30 m. The geometric properties of its cross-section were defined according to the available structural documentation. The bridge girder, sleepers, and rails were represented using beam elements because the study primarily focused on their global and longitudinal displacement responses under moving train loads. This modelling approach provided a suitable compromise between the representation of the track–bridge interaction and the computational efficiency required for repeated FE analyses during optimisation.

The concrete sleepers were connected to the bridge girder using rigid links, allowing the load transferred through each sleeper to be distributed to the corresponding deck locations. The rails were connected to the sleepers through elastic links representing the rail pads and fastening assemblies. The longitudinal spacing of these connections was 630 mm, corresponding to the nominal fastener spacing adopted in the investigated track system. The same spacing was used as the basis for the longitudinal discretisation of the rail and sleeper components. This explicit representation of the rail fasteners was necessary because variations in their stiffness can influence the LVDT responses measured directly on the rails.

**Table 1:** Initial mechanical properties adopted in the finite element model.

Model Component	Property	Unit	Initial Value
Rail P50	Cross-sectional area	cm^2^	77.45
	Second moment of area about the horizontal axis	cm^4^	3217
	Second moment of area about the vertical axis	cm^4^	524
	Elastic modulus	MPa	2.1 × 10^5^
Rail fastening	Vertical fastening stiffness per assembly	kN/mm	35
	Lateral stiffness per assembly	kN/mm	50
Concrete sleeper	Concrete class	-	C50
	Elastic modulus	MPa	3.45 × 10^4^
	Mass density	kg/m^3^	2500
Box girder	Concrete class	-	C50
	Elastic modulus	MPa	3.45 × 10^4^
	Mass density	kg/m^3^	2500

The train-induced response was calculated using linear dynamic time-history analysis with modal superposition, a time step of 0.001 s, and 2% damping in the retained modes. The four-car train was represented by 16 moving axle loads. For this illustrative configuration, lateral wheel–rail forces were assumed to equal 5% of the corresponding vertical axle loads and were applied concurrently at the positions of the moving axles. Each passage was simulated using its corresponding measured speed within the recorded range of 40–80 km/h; 55.6 km/h refers only to the representative passage illustrated in the response plots. Loading inputs were held fixed during each updating run.

The predicted LVDT responses were calculated from the displacement difference between each rail contact point and its corresponding girder-mounted reference:

(12) $${\hat{\delta }}_{i,d}(t)\;=\;e_{d}^{T}\left[u_{P_{i}}(t)\;-\;u_{R_{i}}(t)\right],$$

where Pi denotes the rail contact point, Ri denotes the mounting-reference point, and $e_{d}^{T}$ specifies the measurement direction. The reference displacement represents motion at the actual mounting location, including rigid offsets where applicable. The measured and predicted histories followed the same sign convention, baseline definition, and observation window.

An initial analysis was performed before parameter updating to evaluate the discrepancy between the nominal FE model and the measured displacement records. The initial model reproduced the general sequence of axle-induced response peaks but showed differences in response amplitude and local signal evolution. These discrepancies indicated that the nominal properties did not fully represent the in-service behaviour of the rail-bridge system and justified the measurement-informed updating described in the following section.

The model was intentionally kept at a level of complexity compatible with repeated optimization analyses. Local three-dimensional effects, detailed wheel–rail contact behaviour, and explicit modelling of individual fastening components were not considered. Their combined influence was instead represented through equivalent connection properties. Consequently, the updated parameters should be interpreted as effective model quantities that reproduce the monitored response within the adopted modelling assumptions.

### 3.3. Parameter Selection and Updating Configuration

The selection of updating parameters was based on their expected influence on the measured LVDT responses, uncertainty in their nominal values, and the limited number of available measurement channels. Introducing too many variables could result in a non-unique solution, in which different parameter combinations reproduce similar displacement histories. The updating problem was therefore restricted to three effective parameters representing the global bridge stiffness and the local rail–structure interaction (Table 2).

Local identifiability was assessed at the selected updated solution using the Jacobian of the normalized displacement residuals with respect to dimensionless parameters. Regularization residuals were excluded to distinguish information supplied by the measurements from that imposed by the penalty term. Singular value decomposition was used to assess the conditioning of the response Jacobian. Separately, complete calibration passages were sampled with replacement, and the model was recalibrated for each bootstrap resample using the same objective function, parameter bounds, and optimization settings. The resulting parameter estimates provided percentile-based 95% confidence intervals and pairwise Pearson correlations. This passage-level analysis was distinguished from the independent optimization runs.

The preliminary screening examined each candidate parameter separately, keeping the remaining properties at their nominal values. It identified parameters that affected the predicted responses at the LVDT locations, but did not establish whether the measurements could distinguish their individual effects. Given the limited measurement information, the updating procedure retained three parameters: the box-girder elastic modulus and the effective vertical and lateral fastening stiffnesses.

Different combinations of these parameters may produce similar displacement histories. Both the girder elastic modulus and vertical fastening stiffness can affect vertical rail displacement. Where the measured response includes both girder and fastening deformation, an increase in one stiffness may offset a reduction in the other. The extent of this compensation depends on the LVDT reference and the relative contributions of the two deformation mechanisms. Lateral displacement records provide additional information about lateral fastening behaviour, but do not necessarily distinguish the effects of all three parameters. Displacement histories retain more information than peak values alone, although closely correlated time samples provide limited independent information for parameter identification.

Physical bounds restrict the parameter search, while regularisation penalises departures from the nominal values. These measures help stabilise the solution without establishing that the measurements uniquely determine each parameter. When several parameter combinations fit the data similarly, regularisation favours those with a smaller prescribed penalty. The updated values therefore represent effective model parameters whose estimates depend on the measurement configuration, prescribed train loading, and FE modelling assumptions.

A local sensitivity analysis quantified the effects of the three selected parameters on the predicted LVDT responses. Each parameter was varied by ±1% around its nominal value, while the remaining parameters and loading conditions remained unchanged. The analysis used the same sensor locations, displacement reference, and time window as the calibration procedure. The dimensionless sensitivity index for each response direction was calculated as:

(13) $$I_{\mathrm{dk}}=\frac{1}{2h}\sqrt{\frac{1}{N_{d}}\sum_{r=1}^{N_{d}}{\left(\frac{u_{r,k}^{+}-u_{r,k}^{-}}{s_{r}}\right)}^{2}},\;\;h=0.01,$$

where $u_{r,k}^{+}$ and $u_{r,k}^{-}$ are the predicted displacements following positive and negative perturbations of parameter k, respectively. *N_d_* denotes the number of retained samples in direction d, and *s_r_* is the fixed measured peak amplitude used to normalise the corresponding record. Table 3 summarises the vertical and lateral sensitivity indices. Larger indices indicate greater response sensitivity, although they do not establish that the measurements can distinguish the individual parameter effects.

#### 3.3.1. Data Allocation and Objective-Function Configuration

The valid LVDT records were divided according to complete train-passage events. Seventy percent of the available passages were used for model calibration, while the remaining 30% were retained for independent validation. All samples belonging to the same train passage were assigned to only one group. This event-based division prevented closely correlated portions of a single signal from appearing in both calibration and validation datasets. Calibration and validation used separate train passages from the same monitoring campaign. Validation assesses prediction within the monitored operating conditions; performance under different train configurations, speeds, or temperatures has not been established.

Baseline correction removed the initial offset from each record. The first clearly identifiable axle-related event defined the time reference. The same event-based alignment rule applied to all model predictions for a given passage, without model-specific lag adjustment to minimize the residuals. Measured and predicted responses were compared over identical evaluation intervals. Numerical outputs were interpolated onto the measurement timestamps where required.

The vertical and lateral signals were scaled separately using their maximum measured absolute amplitudes. Equal weights were assigned to the two displacement components because both channels were considered relevant to the behaviour of the rail–bridge system. The objective function was evaluated over the complete retained displacement histories rather than using only their maximum values. It allowed the updating procedure to consider response amplitude, temporal evolution, and the sequence of axle-induced peaks simultaneously.

A regularisation weight of 0.01 was adopted to limit excessive deviations from the nominal parameter values. This relatively small value allowed the measured displacement residuals to dominate the optimization while discouraging unrealistic parameter changes when different solutions produced similar response errors.

#### 3.3.2. Hybrid DE–LM Configuration

The Differential Evolution stage used a population of 30 candidate solutions, corresponding to ten individuals per updating parameter. The initial population was randomly generated within the bounds listed in Table 2 and Table 4. The DE/rand/1/bin strategy was adopted with a mutation factor of 0.70 and a crossover probability of 0.90. These settings provided sufficient population diversity while maintaining stable convergence for the three-dimensional search problem.

**Table 4:** Optimization settings adopted for the hybrid DE–LM procedure.

Algorithm Component	Setting	Adopted Value
DE	Population size	30
	Mutation strategy	DE/rand/1/bin
	Mutation factor	0.7
	Crossover probability	0.9
	Minimum generations before switching	30
	Maximum generations	150
DE-to-LM transition	Monitoring window	10 generations
	Relative-improvement threshold	10^−4^
LM	Initial damping value	0.01
	Finite-difference perturbation	1%
	Objective-function tolerance	10^−6^
	Parameter-increment tolerance	10^−5^
	Maximum iterations	30
Objective function	Regularisation weight	0.01
Repeated analysis	Number of independent runs	20
Comparison	Maximum FE evaluations per run	5000

DE-only and hybrid DE–LM each underwent 20 independent optimization runs using different random seeds. Final calibration objectives were summarized across these runs to assess optimizer variability. A two-sided permutation test compared the mean objectives, and bootstrap resampling of the run-level results provided a 95% confidence interval for their difference. For each method, the parameter set with the lowest calibration objective was selected without reference to validation performance. The validation NRMSE was then calculated separately for each of the 13 withheld training passages. Since both selected models were evaluated on identical passages, their validation errors were compared using a paired permutation test based on within-passage differences. Confidence intervals for the mean validation-error difference were obtained by resampling complete passage pairs.

For comparative analysis, DE alone and LM alone were implemented as reference methods. DE used the same population size, mutation factor, crossover probability, and parameter bounds as the global stage of the hybrid procedure. LM was initialised directly from the nominal parameter values. The same calibration records, objective function, parameter bounds, and stopping budget were used for all algorithms.

Loading sensitivity was assessed by perturbing axle loads and passage speeds separately by ±5%. For each case, the three parameters were recalibrated using unchanged measurement records, parameter bounds, regularization, and optimization settings. In the axle-load cases, vertical and associated lateral forces were scaled together. Parameter changes were calculated relative to the solution obtained with the baseline loading.

After completion of the optimisation, the best parameter set was introduced into the FE model without further adjustment. The updated model was then evaluated using the 30% of train-passage records excluded from calibration. This separation allowed the validation stage to assess the predictive capability of the updated model rather than its ability to reproduce the data used directly during optimisation.

Computational cost was assessed using the total number of FE analyses and elapsed optimization time. The analysis count included initial population evaluations, trial solutions, finite-difference calculations of the LM Jacobian, and rejected trial steps that required an FE solution. For the hybrid method, the count covered both the DE and LM stages. Where one objective-function evaluation required simulations of several train passages, each FE analysis was counted separately. Validation analyses were excluded from the optimization cost and reported separately. All timing comparisons used the same hardware, solver settings, and level of parallel execution.

## 4. Results and Discussion

### 4.1. Initial Model Assessment

The initial FE model was evaluated before structural parameter updating to establish a reference for assessing the effectiveness of the proposed DE–LM procedure. The train speed and axle load identified in the previous field investigation were used as fixed loading inputs. Therefore, the discrepancies discussed in this section primarily reflect uncertainty in the structural and track parameters rather than uncertainty in the operational train characteristics.

**Figure 7:**
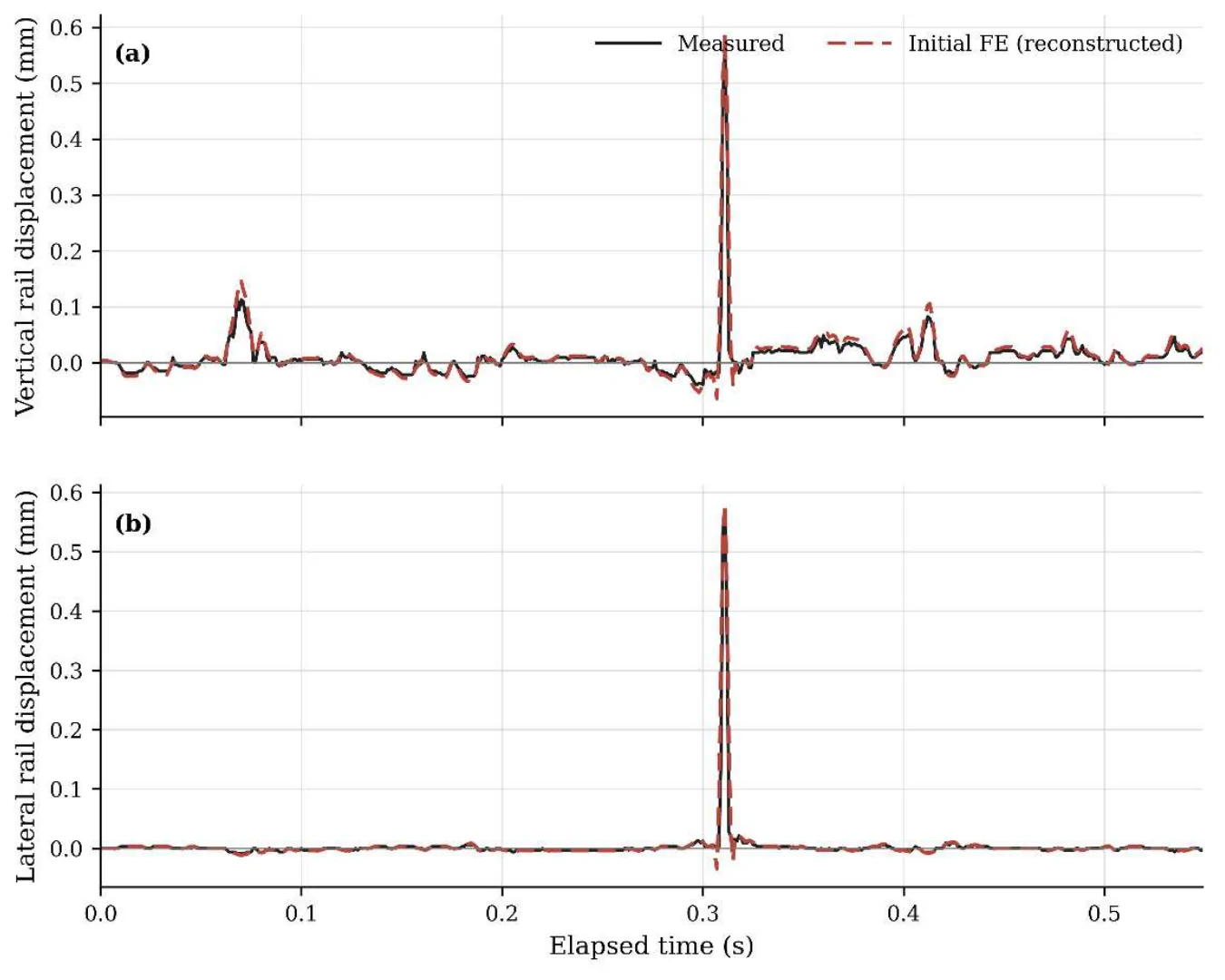
Measured relative rail–girder displacements and predictions from the initial FE model over a selected response interval: (**a**) vertical; (**b**) lateral.

Figure 7 compares the measured and simulated displacement histories at the LVDT locations. The initial model reproduced the overall response pattern associated with the train passage. In particular, the numerical histories exhibited a sequence of peaks corresponding to the successive axles of the four-car train. This agreement indicates that the adopted moving-load sequence, train speed, axle spacing, and temporal synchronisation were generally consistent with the field measurements.

Despite reproducing the general sequence of the measured response, the initial model exhibited differences in peak amplitude and in the local evolution of the displacement histories. The discrepancies were more evident in the vertical response, suggesting that the nominal box-girder and rail-support stiffnesses did not fully represent the effective in-service properties of the monitored system. Smaller differences were observed in the lateral response, although local residuals remained around several axle-induced peaks.

Figure 7 presents a selected interval of the measured and initial-model relative rail–girder displacement histories. The initial-model curves reproduce the principal response peak, although differences remain in its amplitude and local shape. The displayed interval illustrates the local response and does not represent a complete train passage.

**Figure 8:**
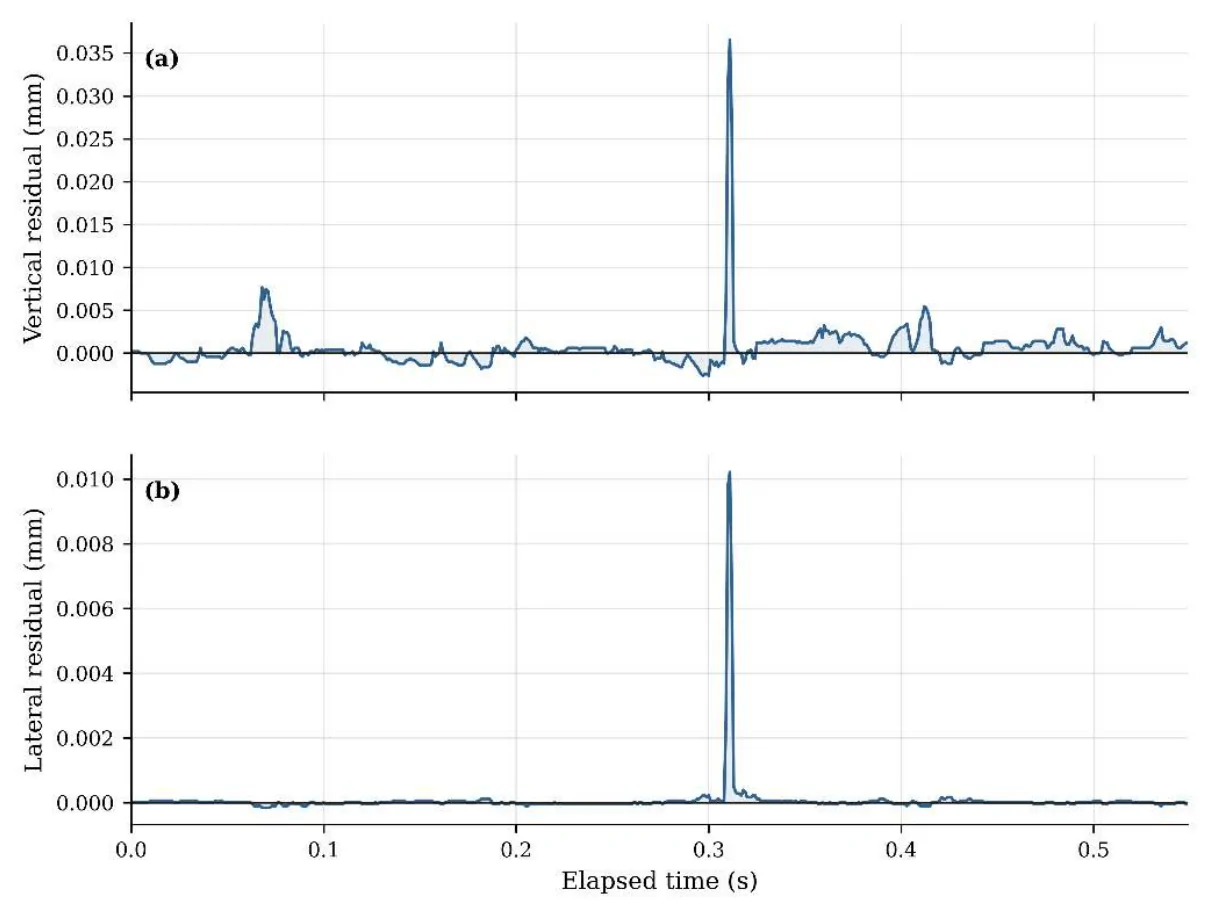
Residual displacement histories of the initial FE model: (**a**) vertical response; and (**b**) lateral response.

Figure 8 shows the corresponding residuals over the same interval. The largest differences occur near the principal response peak, while the remaining portions show smaller deviations.

The initial model captured the principal characteristics of the measured train-induced response but did not provide uniform agreement across the two displacement components and the complete time histories. These remaining differences provided the basis for updating the box-girder elastic modulus and effective fastening stiffnesses using the hybrid DE–LM procedure. The initial results reported in this section were used as the baseline against which convergence, parameter changes, and predictive improvement were evaluated in the following sections.

### 4.2. Convergence and Updated Model Parameters

The convergence performance of the proposed hybrid DE–LM procedure was evaluated against DE-only and LM-only updating. All three methods used the same objective function, calibration records, parameter bounds, and initial FE model. DE-only employed the same population size, mutation factor, and crossover probability as the global stage of the hybrid method. LM-only was initialised directly from the nominal parameter vector, whereas the LM stage of the hybrid method was initialised using the best solution identified by DE.

**Figure 9:**
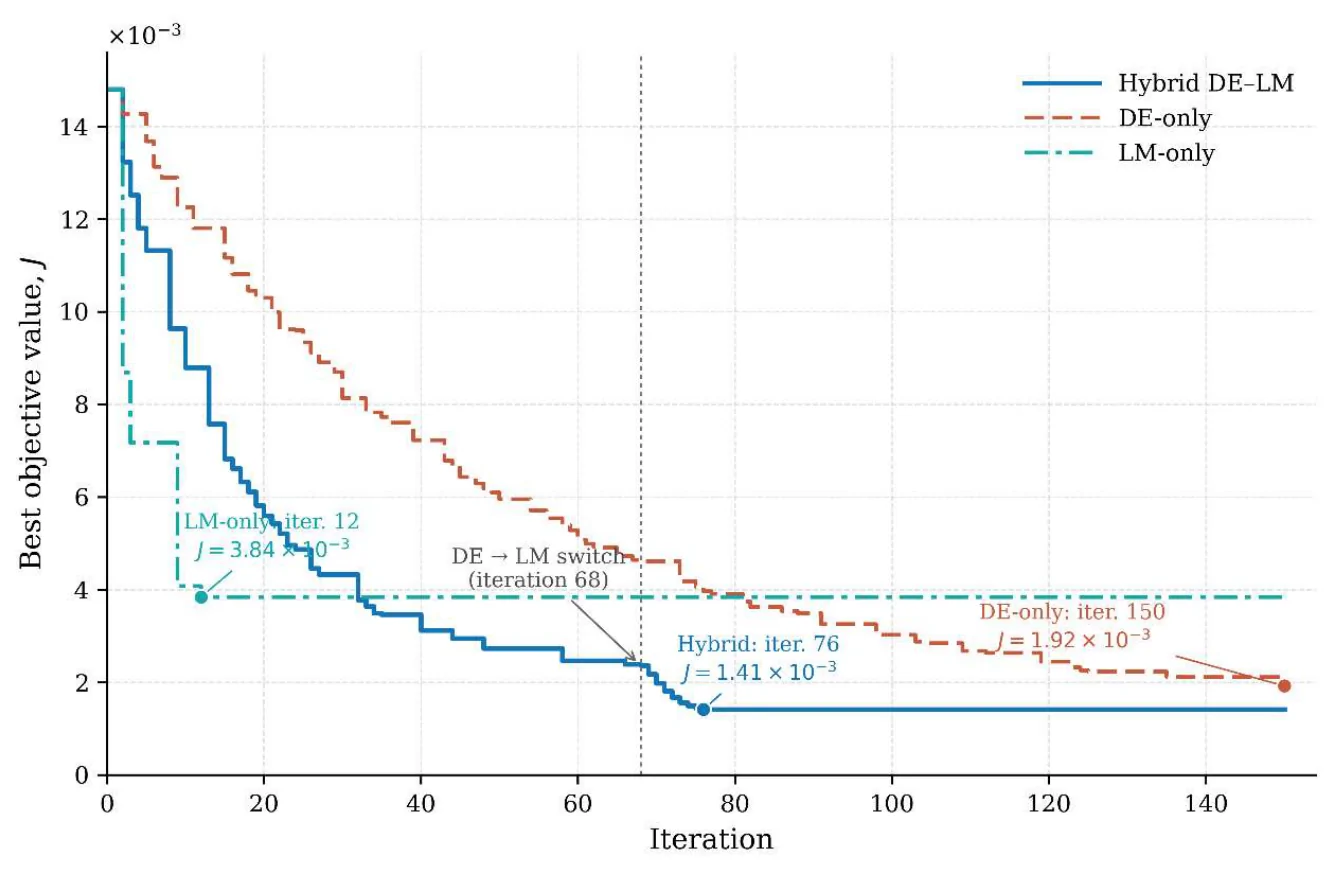
Convergence histories of selected individual DE-only, LM-only, and hybrid DE–LM runs.

Figure 9 compares the convergence histories of the three methods. The objective value of the initial model was 1.48 × 10^−2^. LM-only terminated after 12 iterations with an objective value of 3.84 × 10^−3^, upon satisfying the stopping criterion. This single-start result does not establish the best performance attainable by LM. Dependence on initialization and the adopted 1% finite-difference step remains a limitation of the comparison.

DE-only reached an objective value of 1.92 × 10^−3^ after 150 generations. The objective was still decreasing when the generation limit was reached. This result represents budget-limited termination rather than established convergence.

The hybrid method combined these two convergence characteristics. DE first reduced the objective value to 2.36 × 10^−3^. The switching condition was satisfied after 68 generations, after which the best DE solution was transferred to LM. Local refinement required eight additional iterations and reduced the objective value to 1.41 × 10^−3^. For the selected solutions, the hybrid objective value was 26.6% lower than the DE-only value and 63.3% lower than the LM-only value. The eight LM iterations reduced the objective by 40.3% relative to the switching point, demonstrating effective local refinement. Continued DE optimization might also have produced further improvement. Table 5 summarises the distributions of the final objective values and updated parameters across the independent runs. The statistical comparison uses these distributions rather than the selected solutions alone.

Table 5 distinguishes optimization variability from passage-to-passage prediction variability. The calibration-objective statistics summarize all 20 optimization runs, whereas the validation statistics describe the selected models evaluated on the same 13 withheld passages. The mean vertical and lateral NRMSE differences were 1.14 and 0.65 percentage points, respectively. These validation comparisons assess the predictions of the selected models. Table 6 compares the computational costs of the three methods alongside their final objective values.

The robustness assessment considered all 20 independent DE–LM runs performed with different random seeds under the same calibration data and optimization settings. Table 7 summarises the mean, standard deviation, and range of the final objective values and updated parameters. The objective-value distribution describes the consistency of the final fit, while the parameter distributions indicate whether different runs reach similar parameter combinations. These statistics assess the numerical repeatability of the optimization procedure; they do not quantify uncertainty arising from measurement noise or establish parameter uniqueness.

Table 8 presents the parameters, bootstrap 95% confidence intervals, and pairwise correlations. The Jacobian singular values, scaled by the largest value, were 1.000, 0.420, and 0.065, giving a condition number of 15.38. The correlation between girder modulus and vertical fastening stiffness was −0.86, indicating substantial parameter compensation, whereas correlations involving lateral fastening stiffness were weaker (0.08 and −0.12). Thus, the individual sensitivities in Table 3 do not establish independent identifiability. The bootstrap intervals quantify passage-related variability conditional on the adopted loading and model assumptions, while the 20-run statistics describe numerical repeatability. Accurate response predictions do not establish equally accurate estimates of individual physical properties.

The parameter values obtained using the three methods are presented in Table 9. All solutions remained within the prescribed physical bounds, although the magnitude of the parameter changes varied among the algorithms. LM-only produced smaller changes because its search remained close to the nominal parameter vector. DE-only identified a parameter combination closer to the final hybrid solution, confirming that the global stage had located an appropriate region of the search space. LM subsequently refined this solution to obtain the lowest displacement residual.

The hybrid solution increased the box-girder elastic modulus from 34,500 to 36,810 MPa, corresponding to a change of 6.70%. The effective vertical fastening stiffness increased by 12.57%, from 35.0 to 39.4 kN/mm, while the lateral fastening stiffness increased by 4.20%, from 50.0 to 52.1 kN/mm. These moderate changes are consistent with the initial model assessment, which showed that the nominal model slightly overestimated both vertical and lateral displacements.

The girder-mounted reference limits the interpretation of the estimated elastic modulus. The measurements describe relative rail–girder motion and therefore do not directly constrain global girder deflection. Girder stiffness may still influence the recorded response through the coupled rail–bridge behaviour, but the strength and independence of this influence require quantitative assessment. The reported 6.70% modulus increase is consequently a model-calibration result, rather than an independently verified difference in concrete properties. It may also reflect compensation involving fastening stiffness, uncertainty in prescribed train loading, or modelling assumptions.

The identified fastening stiffnesses represent the equivalent vertical and lateral behaviour of each rail–sleeper connection in the adopted elastic-link model. They describe the combined response of the rail pad, fastening assembly, and associated interfaces, rather than independently measured properties of individual components. Since the model assigns uniform fastening properties along the span, the updated values do not resolve differences between individual connections.

These parameters may also compensate for local deformation and spatial variability that the model does not explicitly represent, as well as simplifications in the sleeper–girder connections and uncertainty in train loading. The estimated girder elastic modulus may similarly reflect both material-property differences and compensation involving the fastening parameters or other modeling assumptions. The changes from nominal to updated values represent calibration adjustments and cannot be attributed entirely to differences in actual structural properties.

Close agreement between predicted and measured responses does not establish that the measurements uniquely determine each parameter. Validation against withheld train passages assesses predictive performance under the operating conditions represented, but does not resolve parameter ambiguity. Likewise, similar solutions across repeated optimization runs would indicate numerical repeatability without proving that the measurements can distinguish the individual parameter values. The updated values should be interpreted as effective model parameters applicable to the investigated measurement and loading conditions.

In sensitivity assessment (Table 10), ±5% axle-load perturbations produced parameter changes of up to 4.70%, whereas speed perturbations produced changes of up to 1.00%. The greater dependence on axle load illustrates potential compensation between prescribed loading and calibrated stiffness.

### 4.3. Model Validation and Comparative Performance

The predictive capability of the updated models was evaluated using the 30% of train-passage records that were excluded from parameter calibration. No additional adjustment of the model parameters, loading conditions, temporal alignment, or scaling factors was permitted during validation. The resulting agreement therefore reflects the ability of each updated model to reproduce independent LVDT responses rather than its fit to the calibration data.

The validation performance was assessed using the normalized root mean square error (NRMSE), coefficient of determination, and relative peak-displacement error. NRMSE was normalized by the maximum absolute measured displacement within the evaluation interval:

(14) $$\begin{array}{l} \mathrm{NRMSE}=\frac{100}{\max_{j}|y_{j}|}\sqrt{\frac{1}{N}\sum_{j=1}^{N}{\left({\hat{y}}_{j}-y_{j}\right)}^{2}},\;\;\\ R^{2}=1-\frac{\sum_{j=1}^{N}{\left(y_{j}-{\hat{y}}_{j}\right)}^{2}}{\sum_{j=1}^{N}{\left(y_{j}-\bar{y}\right)}^{2},} \end{array}$$

Both metrics were calculated for each passage and measurement channel. Since a narrow dominant peak can strongly influence R2, this metric was interpreted alongside NRMSE and peak errors. Shifting the predicted histories by ±1 sample (±0.01 s), without recalibration, increased the mean validation NRMSE from 2.58% to 3.48–3.64% vertically and from 1.76% to 2.94–3.10% laterally. All comparisons used a common overlapping interval and unchanged normalization, indicating sensitivity to temporal alignment around the narrow peaks.

NRMSE summarises the discrepancy between measured and predicted displacements over the evaluation interval. The peak error was included as an engineering indicator of the model’s ability to reproduce the maximum train-induced displacement.

**Figure 10:**
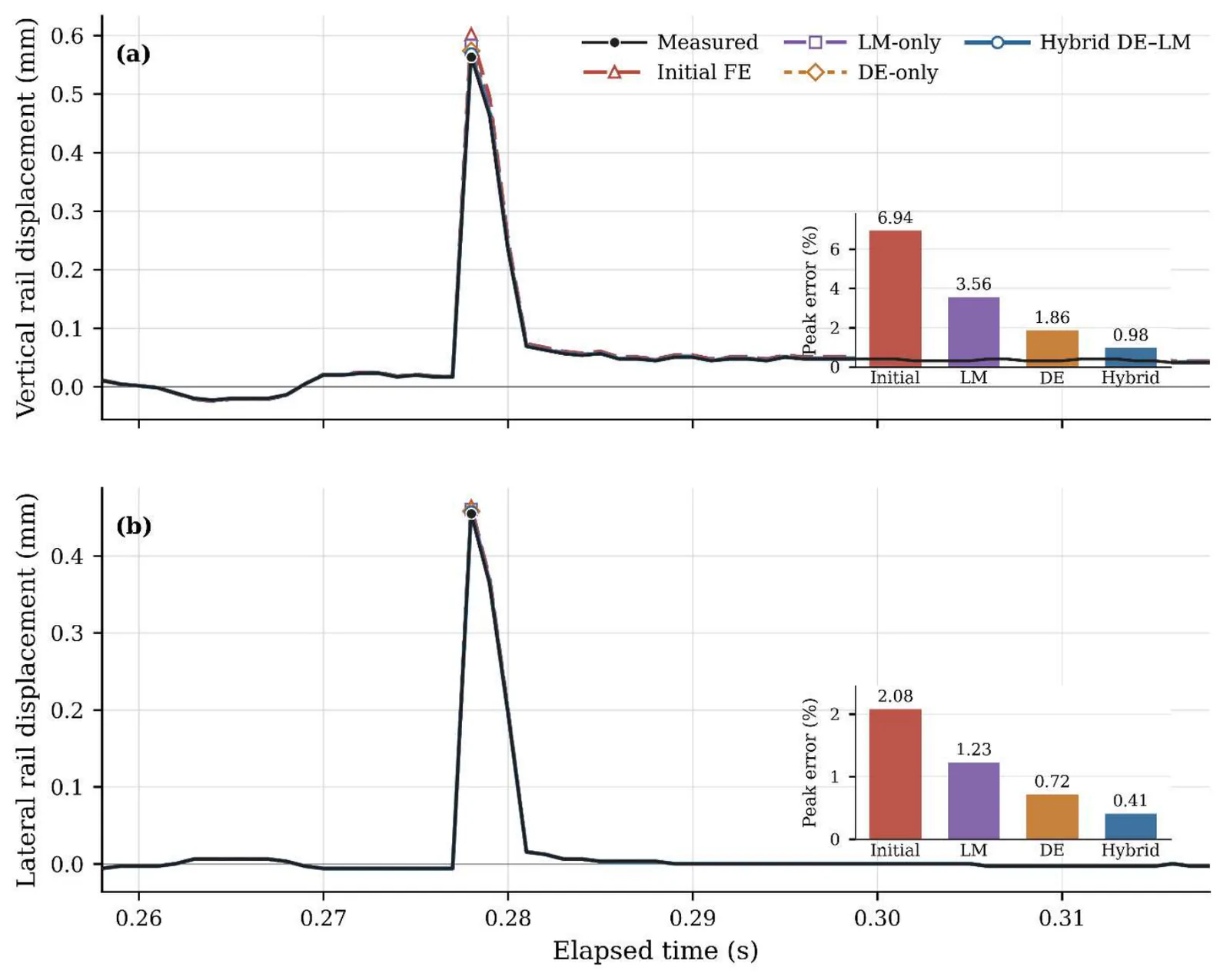
Enlarged validation response segment comparing measurements with predictions from the initial and updated models: (**a**) vertical; (**b**) lateral.

Figure 10 presents an enlarged segment of a withheld validation record. The curves illustrate differences in peak amplitude and local response shape between the measurements and the initial and updated model predictions. The displayed segment provides a detailed comparison around one response peak.

The quantitative validation results are summarised in Table 11. For the vertical response, the initial model produced an NRMSE of 9.81%, which decreased to 6.21% using LM-only, 3.72% using DE-only, and 2.58% using the hybrid method. The corresponding coefficient of determination increased from 0.913 for the initial model to 0.987 for the hybrid model. A similar improvement was observed for the lateral response, for which the hybrid method achieved an NRMSE of 1.76% and a coefficient of determination of 0.990.

**Figure 11:**
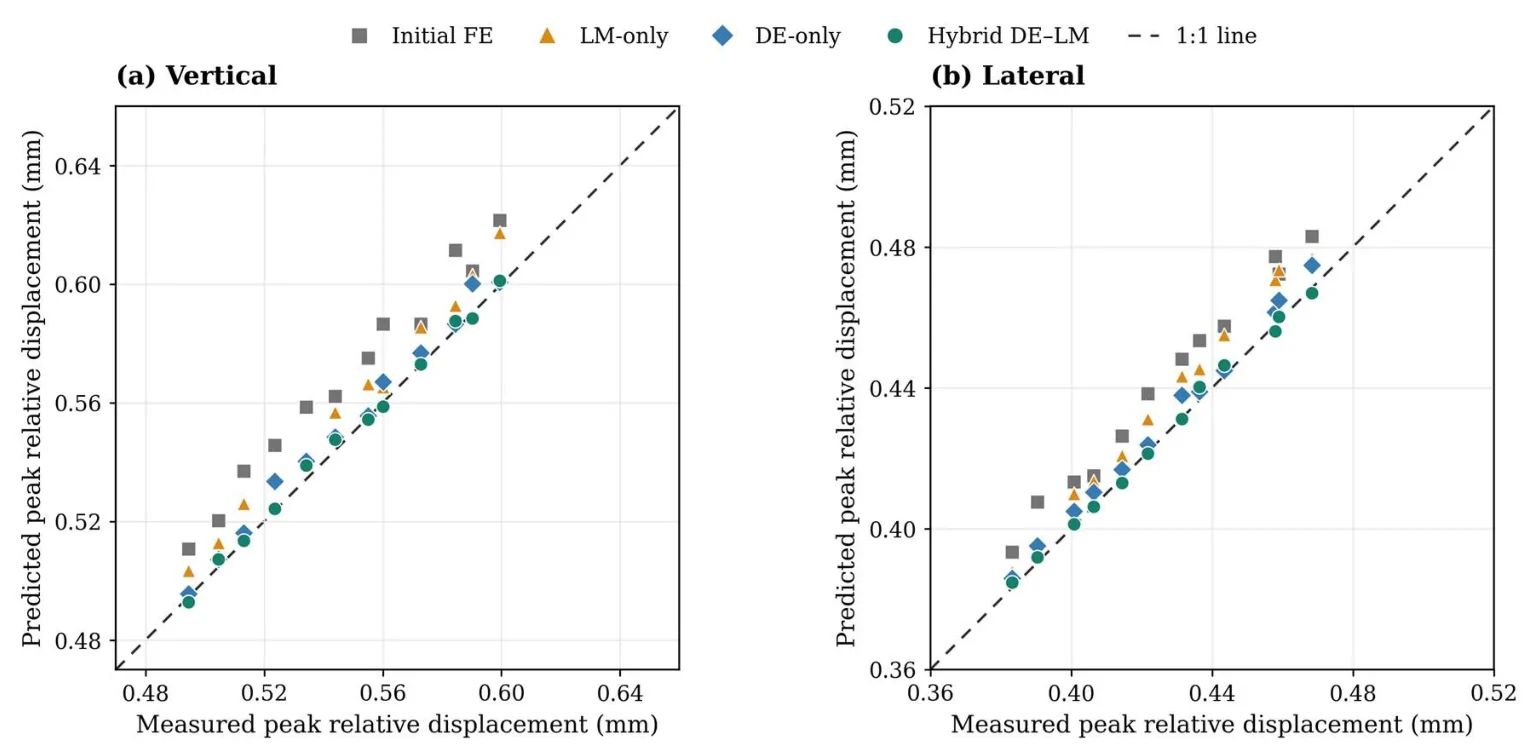
Measured versus predicted peak relative displacements across all validation passages: (**a**) vertical; (**b**) lateral.

Figure 11 shows closer agreement between measured and predicted peak relative displacements after model updating. The initial model overestimates both response components, while the hybrid DE–LM predictions lie closest to the equality line for most passages.

The selected DE-only and hybrid solutions differ by approximately 0.25% in girder elastic modulus and 1.55% in vertical fastening stiffness, while their vertical NRMSE values differ by 1.14 percentage points. These differences alone do not establish whether the predictive improvement exceeds optimization variability or data-related uncertainty. The three parameters change jointly, so the difference in response cannot be attributed to a single parameter. The repeated-run statistics and passage-level confidence intervals provide the basis for interpreting this improvement.

**Figure 12:**
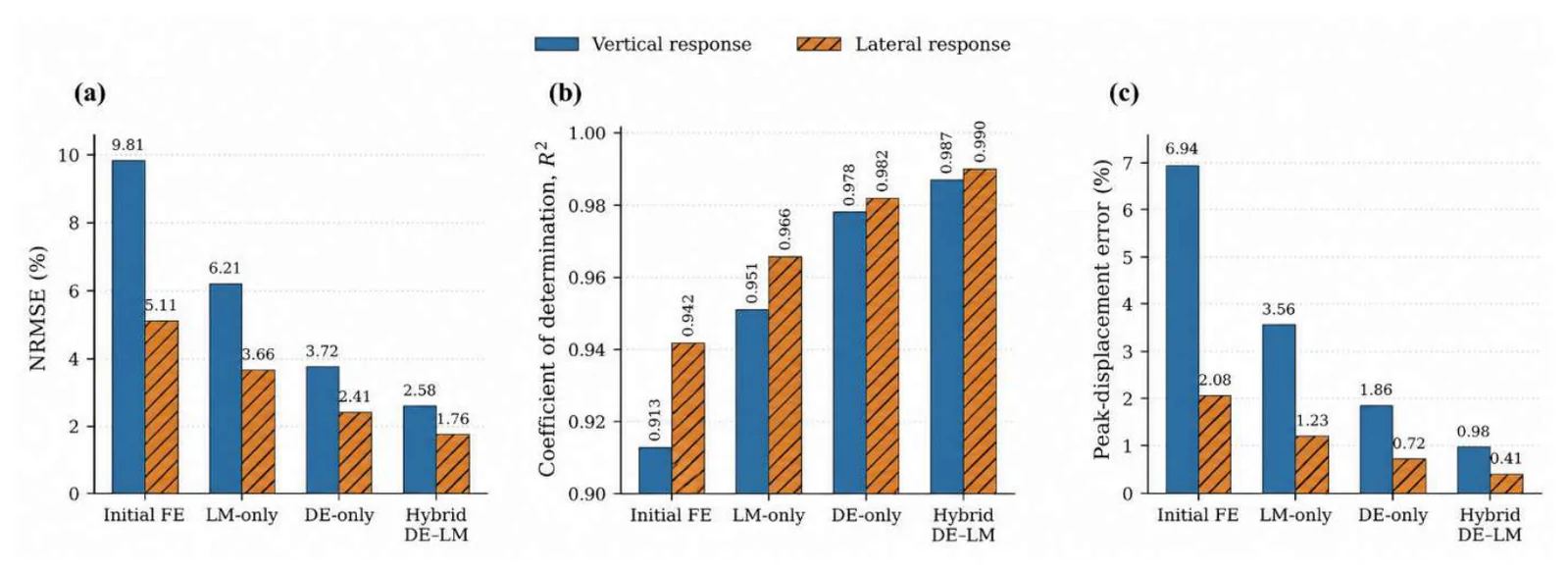
Comparative validation performance of the initial, LM-only, DE-only, and hybrid DE–LM models in terms of (**a**) NRMSE, (**b**) coefficient of determination, and (**c**) peak-displacement error.

The validation results show that the selected hybrid model produced lower displacement prediction errors than the initial model on withheld train passages, with closer agreement in peak displacements, as illustrated in Figure 11. Averaged over the two response components, the hybrid method reduced NRMSE from 7.46% for the initial model to 2.17%. In comparison, the mean NRMSE values obtained using LM-only and DE-only were 4.94% and 3.07%, respectively. The hybrid model also reduced the mean peak error to 0.70%, compared with 1.29% for DE-only and 2.40% for LM-only (Figure 12).

The selected hybrid solution produced lower validation errors than the DE-only and LM-initialized solutions from the nominal parameter vector. These results support the effectiveness of the sequential procedure for the investigated case. However, the comparison does not establish whether a comparable result could be obtained with LM using multiple initializations within the same computational budget.

The validation results show that the updated model can reproduce measured responses from train passages excluded from calibration. This provides evidence of predictive performance within the monitored operating regime. However, the similarity of the train configuration and the lack of documented contrasts in passage-specific loading and environmental conditions limit the scope of this assessment. The results do not establish performance under different train compositions, substantially different speeds, or seasonal temperature variations. Such conditions require separate validation before the model can support a broader range of operational applications.

Residual differences were still observed around several axle passages, particularly in the vertical response. These differences may arise from the simplified moving-load representation, variations in axle loads among train passages, local wheel–rail irregularities, uncertainty in the operational speed, measurement noise, and the assumption of uniform fastening stiffness along the span. The updated properties should therefore be interpreted as effective parameters of the adopted rail–bridge model rather than direct estimates of individual material or fastening properties.

Measurement uncertainty arises from LVDT calibration and resolution, acquisition noise, baseline drift, sensor alignment, and movement of the supporting frame or reference point. These effects become more significant relative to smaller displacement responses. Baseline correction removes initial offsets but cannot correct reference movement during train passage. Timing errors may also increase residuals near narrow response peaks. Peak normalization scales both the response and its noise, while equal channel weights do not imply equal measurement precision. The reported errors indicate agreement with the recorded signals. Differences of approximately 0.001 mm do not demonstrate comparable physical accuracy without a quantified uncertainty budget for the complete measurement system. Measurement uncertainty may also influence the updated parameters, particularly when different parameter combinations produce similar responses.

The proposed framework provides a calibrated FE model as the physics-based component of a railway-bridge digital twin. The present implementation uses previously recorded LVDT measurements for offline parameter estimation and evaluates the resulting model against withheld train passages. This process establishes a connection between field observations and the numerical model but does not demonstrate continuous data assimilation or automated model updating. Further development would require a persistent connection to the monitoring system, automated data-quality checks, and procedures for determining when and how new measurements should trigger parameter updates. Such procedures would also need to distinguish structural changes from variations in loading, environmental conditions, and measurement uncertainty. The present contribution is therefore limited to baseline model calibration and prediction under the monitored conditions. Continuous synchronization, automated condition assessment, and maintenance decision support remain beyond the demonstrated scope.

## 5. Conclusions

This study proposed a hybrid DE–LM framework for updating an FE model of an urban railway bridge using train-induced LVDT displacement histories. DE provided global exploration, while LM efficiently refined the most promising solution. The updated parameters remained physically admissible and reduced the initial objective value by approximately 90.5%.

For the monitored passages, hybrid DE–LM achieved lower calibration and validation errors than the reported DE-only and LM-only solutions. It achieved a mean validation NRMSE of 2.17%, compared with 3.07% for DE-only, 4.94% for LM-only, and 7.46% for the initial model. The updated model provides a calibrated reference for the measured responses within the investigated conditions. These findings are conditional on the adopted initialization, stopping criteria, and computational budget.

The findings are limited to one span, one monitoring campaign, one train type, and four measurement channels. The three updated parameters only represent effective model properties. Further assessment should include different operating conditions and controlled stiffness changes to establish whether the framework can distinguish structural changes from measurement and loading variability.

## Figures and Tables

**Table 2:** Parameters selected for finite element model updating.

Updating Parameter	Unit	Initial Value	Lower Bound	Upper Bound	Main Response Influenced
Box-girder elastic modulus	MPa	34,500	27,600	41,400	Contribution to relative rail–girder response through coupled structural behaviour
Effective vertical fastening stiffness	kN/mm	35.0	17.5	52.5	Vertical relative rail–girder displacement
Effective lateral fastening stiffness	kN/mm	50.0	25.0	75.0	Lateral relative rail–girder displacement

**Table 3:** Dimensionless sensitivity indices of the selected updating parameters.

Updating Parameter	Vertical Response	Lateral Response
Box-girder elastic modulus	0.19	0.01
Effective vertical fastening stiffness	0.43	0.02
Effective lateral fastening stiffness	0.05	0.35

**Table 5:** Comparison of calibration objectives across independent optimization runs and prediction errors across withheld validation passages.

Performance Metric	DE-Only, Mean ± SD	Hybrid DE–LM, Mean ± SD	Basis of Calculation
Final calibration objective	1.973 ± 0.036 × 10^−3^	(1.444 ± 0.023) ×10^−3^	20 independent optimization runs per method
Vertical validation NRMSE (%)	3.72 ± 1.36	2.58 ± 0.55	13 withheld train passages
Lateral validation NRMSE (%)	2.41 ± 1.28	1.76 ± 0.45	13 withheld train passages

**Table 6:** Computational costs and final calibration objectives of the selected optimization runs.

Method	Optimization Steps Reported	Estimated FE Analyses per Run	Final Objective Reported
LM-only	12 LM iterations	49	3.84 × 10^−3^
DE-only	150 DE generations	4530	1.92 × 10^−3^
Hybrid DE–LM	68 DE generations + 8 LM iterations	2102	1.41 × 10^−3^

**Table 7:** Statistical summary of the hybrid DE–LM results over 20 independent runs.

Quantity	Mean	Standard Deviation	Minimum	Maximum
Final objective value	1.442 × 10^−3^	1.925 × 10^−5^	1.410 × 10^−3^	1.485 × 10^−3^
Box-girder elastic modulus (MPa)	36,803.1	39.3	36,718.5	36,866.9
Effective vertical fastening stiffness (kN/mm)	39.425	0.064	39.275	39.553
Effective lateral fastening stiffness (kN/mm)	52.096	0.087	51.949	52.299
Total FE analyses per run	2137.0	61.0	2020	2226

**Table 8:** Parameter estimates, passage-bootstrap 95% confidence intervals, and pairwise correlations.

Parameter	Selected Estimate	Bootstrap 95% CI	Correlation with E_girder_	Correlation with Kv	Correlation with K_lat_
E_girder_ (MPa)	36,810	[35,050; 38,600]	1.00	−0.86	0.08
Kv (kN/mm)	39.4	[37.6; 41.2]	−0.86	1.00	−0.12
K_lat_ (kN/mm)	52.1	[51.1; 53.1]	0.08	−0.12	1.00

**Table 9:** Initial and updated model parameters obtained using the investigated optimization methods.

Updating Parameter	Unit	Initial Value	LM-Only	DE-Only	Hybrid DE–LM
Box-girder elastic modulus	MPa	34,500	35,920	36,720	36,810
Effective vertical fastening stiffness	kN/mm	35.0	37.1	38.8	39.4
Effective lateral fastening stiffness	kN/mm	50.0	51.2	52.5	52.1

**Table 10:** Parameter changes under separate perturbations of axle load and train speed.

Loading Assumption	Change in E_girder_	Change in Kv (%)	Change in K_lat_ (%)
Baseline	0.00	0.00	0.00
Axle loads −5%	−1.80	−4.20	−4.60
Axle loads +5%	+1.90	+4.30	+4.70
Passage speeds −5%	−0.60	−0.90	−0.70
Passage speeds +5%	+0.70	+1.00	+0.80

**Table 11:** Performance of the initial and updated models on the independent validation dataset.

Response Component	Performance Metric	Initial FE	LM-Only	DE-Only	Hybrid DE–LM
Vertical	NRMSE (%)	9.81	6.21	3.72	2.58
	Coefficient of determination	0.913	0.951	0.978	0.987
	Peak error (%)	6.94	3.56	1.86	0.98
Lateral	NRMSE (%)	5.11	3.66	2.41	1.76
	Coefficient of determination	0.942	0.966	0.982	0.99
	Peak error (%)	2.08	1.23	0.72	0.41

## Data Availability

The data presented in this study are available upon request to the corresponding authors due to the host institution’s rules.
